# Clinical validation and utility of Percepta GSC for the evaluation of lung cancer

**DOI:** 10.1371/journal.pone.0268567

**Published:** 2022-07-13

**Authors:** Peter Mazzone, Travis Dotson, Momen M. Wahidi, Michael Bernstein, Hans J. Lee, David Feller Kopman, Lonny Yarmus, Duncan Whitney, Christopher Stevenson, Jianghan Qu, Marla Johnson, P. Sean Walsh, Jing Huang, Lori R. Lofaro, Sangeeta M. Bhorade, Giulia C. Kennedy, Avrum Spira, M. Patricia Rivera, The AEGIS Study Team, The Percepta Registry Investigators

**Affiliations:** 1 Department of Pulmonary Medicine, Cleveland Clinic, Respiratory Institute, Cleveland, OH, United States of America; 2 Division of Pulmonary and Critical Care, Wake Forest Baptist Health, Winston-Salem, NC, United States of America; 3 Division of Pulmonary, Allergy & Critical Care Medicine, Duke University Medical Center, Durham, NC, United States of America; 4 Stamford Health Medical Group, Pulmonary, Stamford Hospital, Stamford, CT, United States of America; 5 Division of Pulmonary and Critical Care Medicine, Johns Hopkins University, Baltimore, MD, United States of America; 6 Head of Early Detection Lung Cancer Initiative, Johnson & Johnson, Boston, MA, United States of America; 7 Head of Pharmaceutical Sciences, Lung Cancer Initiative, Johnson & Johnson, London, United Kingdom; 8 Research and Development, Veracyte, Inc, San Francisco, CA, United States of America; 9 Clinical Operations, Veracyte, Inc, San Francisco, CA, United States of America; 10 Medical Affairs, Veracyte, Inc, San Francisco, CA, United States of America; 11 Research and Development, Clinical Operations, Medical Affairs, Veracyte, Inc, South San Francisco, CA, United States of America; 12 Division of Pulmonary and Critical Care Medicine, Boston University Medical Center, Boston, MA, United States of America; 13 Division of Pulmonary and Critical Care Medicine, University of North Carolina at Chapel Hill, Chapel Hill, NC, United States of America; Inha University Hospital, REPUBLIC OF KOREA

## Abstract

The Percepta Genomic Sequencing Classifier (GSC) was developed to up-classify as well as down-classify the risk of malignancy for lung lesions when bronchoscopy is non-diagnostic. We evaluated the performance of Percepta GSC in risk re-classification of indeterminate lung lesions. This multicenter study included individuals who currently or formerly smoked undergoing bronchoscopy for suspected lung cancer from the AEGIS I/ II cohorts and the Percepta Registry. The classifier was measured in normal-appearing bronchial epithelium from bronchial brushings. The sensitivity, specificity, and predictive values were calculated using predefined thresholds. The ability of the classifier to decrease unnecessary invasive procedures was estimated. A set of 412 patients were included in the validation (prevalence of malignancy was 39.6%). Overall, 29% of intermediate-risk lung lesions were down-classified to low-risk with a 91.0% negative predictive value (NPV) and 12.2% of intermediate-risk lesions were up-classified to high-risk with a 65.4% positive predictive value (PPV). In addition, 54.5% of low-risk lesions were down-classified to very low risk with >99% NPV and 27.3% of high-risk lesions were up-classified to very high risk with a 91.5% PPV. If the classifier results were used in nodule management, 50% of patients with benign lesions and 29% of patients with malignant lesions undergoing additional invasive procedures could have avoided these procedures. The Percepta GSC is highly accurate as both a rule-out and rule-in test. This high accuracy of risk re-classification may lead to improved management of lung lesions.

## Introduction

The diagnosis of screen and incidentally detected lung lesions can be challenging [1–4]. Current guidelines recommend that these lesions be managed based upon their probability of malignancy [5–13]. Patients with lesions having intermediate risk of malignancy present the biggest diagnostic challenge. Management may include continued imaging surveillance, invasive diagnostic procedures, or surgical resection. Bronchoscopy has a low diagnostic yield for smaller or peripherally located lesions [14–17], thus complementary noninvasive diagnostic testing that further stratifies patients may assist in subsequent management decisions and reduce patients’ exposure to unnecessary invasive procedures [18–20].

The first generation Percepta Bronchial Genomic Classifier (BGC), developed using microarray-based gene expression technology, assesses cancer-associated gene expression in cytologically normal-appearing bronchial epithelial cells in the mainstem bronchus [21–23]. This classifier was designed to be a “rule-out” test for intermediate-risk patients with high sensitivity to detect malignancy, allowing their pre-test cancer risk to be re-classified to low (post-test) risk with a non-diagnostic bronchoscopy and a negative test result. Accuracy of the classifier was validated in two large observational multicenter studies, and the utility of the classifier was shown in a multicenter “real world” study [24–26]. In this “real world” prospective multicenter study, a negative Percepta result impacted clinical decision making by down-classifying a significant number of patients with an intermediate risk of malignancy, resulting in a change in clinical management from an invasive procedure to surveillance of the lung lesion [25].

The Percepta Genomic Sequencing Classifier (GSC) is an enhanced second-generation classifier prospectively developed using a testing platform with richer genomic features from whole transcriptome RNA sequencing in combination with clinical factors [27,28]. Analytical validation of Percepta GSC showed reproducibility of the test results in several clinical conditions, including sample collection, storage, shipping, and laboratory processing attesting to the robustness of the classifier across several technical variables [28]. In addition, the Percepta GSC was developed with multiple thresholds allowing it to serve as both a “rule-in” test and a “rule-out” test, thereby increasing its potential utility in improving risk stratification [27]. This study was designed to clinically validate the accuracy of the Percepta GSC in patients with lung lesions who had a non-diagnostic bronchoscopy.

## Methods

### Study design

Patients with an indeterminate lung lesion who had a non-diagnostic bronchoscopy from three different cohorts were evaluated for inclusion. The Airway Epithelium Gene Expression in the Diagnosis of Lung Cancer cohorts (AEGIS I and II) were recruited from multicenter prospective observational studies. Participants were included from 24 centers in the United States, Canada, and Ireland (S1 Fig) if they currently or formerly smoked and were undergoing bronchoscopy to evaluate lung nodules. The Percepta Registry cohort was a multicenter prospective registry that included patients with lung nodules who underwent clinically indicated diagnostic bronchoscopy at 34 medical centers across the US (S2 Fig). Each institution obtained institutional review board (IRB) (Percepta Registry: WIRB# 20151039 and the AEGIS cohorts: IRB #20090312) approval before enrollment, and informed consent was obtained from all patients. Two bronchial brushings were performed during bronchoscopy, and mRNA was collected from bronchial epithelial cells from the right mainstem bronchus. Before bronchoscopy, physicians assessed the risk of malignancy (ROM) for each patient, designated as low (<10%), intermediate (10–60%), or high (>60%) [5]. Study personnel recorded lesion characteristics from the site radiologist report at each institution. In addition, baseline demographic data, smoking history, and subsequent procedures including bronchoscopy, transthoracic needle aspiration/ biopsy, and surgical procedures were recorded. All patients were followed for at least 12 months after bronchoscopy unless a diagnosis of malignancy was confirmed.

### Patient selection

Patients from the AEGIS cohorts and the Percepta Registry were randomly split into training and validation cohorts (S1 and S2 Figs). The previously described algorithm development process was restricted to the training cohort [27]. The algorithm development team was blinded to the validation cohort. Exclusion criteria included age < 21 years old, inability to provide informed consent, lack of tobacco use (smoked < 100 cigarettes), or prior or concurrent cancer history. All patients underwent an adjudication process described below, to determine if the leson was benign or malignant. Forty-five patients from the Percepta Registry who underwent adjudication and had stable imaging after 12 months but did not yet have a confirmed diagnosis were labeled “clinically benign” and excluded from the calculation of sensitivity and specificity of the Percepta GSC validation performance as they did not have individual truth labels. However, given the concern for significant bias of overestimation of cancer prevalence, these “clinically benign” lesions were included in calculating cancer prevalence. (S3 Fig).

### Adjudication of diagnoses (Benign versus malignant lesions)

Diagnosis of a benign or malignant lesion was determined through an adjudication process. For the Percepta Registry Cohort, a live adjudication process was conducted to arbitrate a benign, malignant, or inconclusive consensus diagnosis by an expert 3-member pulmonologist panel. (HJL, DFK, LY). Panel members were provided with de-identified patient information with at least 12 months of follow-up. Members of the panel were blinded to the Percepta GSC results.

A benign diagnosis was assigned in cases with 1) resolution of the lesion; 2) an alternative benign diagnosis; 3) lesion stability for ≥ 12 months and determination by the panel that the patient has no further suspicion of malignancy. Although two-year stability for radiographic imaging of lesions is recommended, this study included one-year follow-up of the lesion based upon prior studies that have found one-year nodule stability to be predictive of stability at two years [1,2]. A malignant diagnosis was assigned in cases with a pathology report confirming malignancy or a decision to treat a patient with stereotactic body radiation therapy (SBRT) without tissue confirmation.

To enhance confidence in the adjudication process, a subset of adjudicated patients underwent a second blinded independent central review by two independent oncologists with adjudication by a third oncologist, if needed. Reviewers were provided with the same clinical information as provided in the first adjudication process. Results were 95% concordant (Cohen’s kappa = 0.88); therefore data from the first adjudication was used for analysis.

The adjudication process for the AEGIS I and II cohorts was performed as previously described [24].

### Evaluation of the validation performance and other statistical analysis

This independent validation set comprised 412 patients with lesions that included low, intermediate, and high pre-test ROM. Descriptive statistics are reported for clinical demographic data by cohort, with differences between cohorts tested with the chi-square test for categorical variables and Wilcoxon rank test for continuous variables. All confidence intervals are two-sided 95% unless otherwise noted. Statistical analyses were performed in R (version 3.2.3, https://www.r-project.org). Performance of the classifier was first assessed with pre-specified cut-offs and associated 2x2 tables to calculate sensitivity, specificity, NPV, and PPV. Performance was also assessed independently of thresholds utilizing a receiver operating curve (ROC) and calculating the area under the curve (AUC). The ROC provided a comprehensive evaluation of the Percepta GSC classifier performance across all three cohorts and in different pre-test ROM groups. (S4 Fig).

### Estimation of the potential impact of Percepta GSC on clinical management of indeterminate lung lesions

The potential impact of Percepta GSC on the number of invasive diagnostic procedures was assessed for patients in the AEGIS I and II cohorts. Patients in the Percepta Registry were excluded from this analysis due to the inclusion of the Percepta classifier in the clinical management of these lesions. The number of invasive procedures in patients with pre-test low- or intermediate- risk benign lesions who were down-classified by the Percepta GSC was calculated to determine the procedures that could have been avoided if the classifier result had been utilized during lesion management. Likewise, patients with pre-test intermediate or high-risk Stage I or II malignant lesions who were up-classified by Percepta GSC and had an intervening diagnostic procedure or underwent surveillance prior to a definitive surgical procedure were assessed to determine if intervening procedures could have been avoided, thereby enabling an earlier diagnosis of malignancy.

## Results

### Clinical study population and lesion characteristics

Four hundred twelve patients from the AEGIS cohorts (I and II) (246 patients) and the Percepta Registry (166 patients) were included in the validation cohort for the Percepta GSC (Table 1, S1 and S2 Figs). The most common histological types of cancer were adenocarcinoma (51%) followed by squamous cell (22%) lung cancer.

**Table 1:** Demographic and clinical characteristics of the study participants.

Characteristic			AEGIS (N = 246)	Registry (N = 166)	Total (N = 412)	P-value
**Sex**						**0.001**
	Female		83 (34%)	84 (51%)	167 (40%)	
	Male		163 (66%)	82 (49%)	245 (59%)	
**Median age (IQR)**			62 (54–70)	65 (58–71)	63 (56–71)	0.08
**Race**						0.38
	White		192 (78%)	132 (80%)	324 (79%)	
	Black		42 (17%)	29 (17%)	71 (17%)	
	Other		12 (5%)	4 (2%)	16 (4%)	
	Unknown		0	1 (1%)	1 (0.2%)	
**Smoking status**						0.92
	Current		107 (43%)	73 (44%)	180 (44%)	
	Former		139 (57%)	93 (56%)	232 (56%)	
**Median cumulative tobacco use (IQR)** –pack-year			35 (20–56)	35 (20–50)	35 (20–54)	0.89
**Lesion size**						**< 0.001**
	Infiltrate*		25 (10%)	0	25 (6%)	
	< 2 cm		88 (36%)	80 (48%)	168 (41%)	
	2 to 3 cm		48 (20%)	29 (17%)	77 (19%)	
	> 3 cm		75 (30%	44 (27%)	119 (29%)	
	Unknown		10 (4%)	13 (8%)	23 (6%)	
**Lesion location**						**< 0.001**
	Central		72 (29%)	10 (6%)	82 (20%)	
	Peripheral		108 (44%)	144 (87%)	252 (61%)	
	Central and peripheral		53 (22%)	0	53 (13%)	
	Unknown		13 (5%)	12 (7%)	25 (6%)	
**Lung-cancer histologic type**			**111 (45%)**	**52** **(31%)**	**163 (40%)**	**0.01**
	Small cell lung cancer		8 (7%)	1 (2%)	9 (6%)	
	Non-small cell lung cancer		100 (90%)	43 (83%)	143 (88%)	0.43
		Adenocarcinoma	58 (58%)	25 (58%)	83 (58%)	
		Squamous	26 (26%)	10 (23%)	36 (25%)	
		Large-cell	4 (4%)	0	4 (3%)	
		NSCLC-NOS	12 (12%)	8 (19%)	20 (14%)	
	Carcinoid		0	2 (4%)	2 (1%)	
	Unknown		3 (3%)	6 (12%)	9 (6%)	
**Diagnosis of a benign condition**			**135 (55%)**	**114 (69%)**	**249 (60%)**	**< 0.001**
	Granuloma		26 (19%)	10 (9%)	36 (14%)	
	Infection		36 (27%)	15 (13%)	51 (20%)	
	Two or more benign conditions		8 (6%)	0	8 (3%)	
	Other		27 (20%)	4 (4%)	31 (12%)	
	Resolution of Stability		38 (28%)	40 (35%)	78 (31%)	
	Clinically benign**		0	45 (39%)	45 (18%)	

IQR, interquartile range; NSCLC-NOS, non-small cell lung cancer- not otherwise specified.

Percentages are calculated within each study cohort, i.e. AEGIS, and the Percepta Registry, respectively; for sub-level breakdowns, i.e. cancer histologic subtype and benign condition, the denominator is the sub-group count.

*Infiltrates are pulmonary lesions with ill-defined margins and a diameter that cannot be accurately defined.

** Clinically benign did not have an adjudicated diagnosis but were included in the analysis for cancer prevalence to prevent an over-estimate.

### Performance of Percepta GSC in indeterminate lesions stratified by risk of malignancy

Pre-test ROM defined approximately 19% of the cohort as low risk (cancer prevalence of 5.0%), 46% as intermediate risk (cancer prevalence of 28.2%), and 35% as high risk (cancer prevalence of 74.0%). Intermediate-risk nodules were down-classified to low-risk with a sensitivity of 90.6% and a specificity of 37.3% by Percepta GSC. With a 28.2% cancer prevalence, 29.4% of intermediate-risk lesions were down-classified with a 91.0% (Confidence Interval (CI): 80.8–96.0) NPV. Intermediate-risk lesions were up-classified to high risk with a 94.1% specificity and 28.3% sensitivity by Percepta GSC. With a 28.2% cancer prevalence, 12.2% of intermediate-risk lesions were up-classified with a 65.4% (CI: 43.8–82.1) PPV. Low-risk lesions were further down-classified to very low risk in 54.5% of cases with a 100% sensitivity, indicating no false negatives and > 99% NPV (CI: 91.0–100). High-risk lesions were up-classified to very high risk, with a specificity of 91.2% and a sensitivity of 34.0%. With a 73.6% cancer prevalence, 27.3% of high-risk lesions were up-classified with a 91.5% (CI: 77.9–97.0) PPV (Table 2).

**Table 2:** Percepta GSC performance.

Pre-test Risk of Malignancy (cancer prevalence)	Malignant	Benign	Clinical Benign	Specificity	Sensitivity	Percepta GSC result	Post-test NPV/PPV	% Re-classified risk of malignancy
Low N = 80 (5.0%)	4	68	8	57.4% (44.8–69.3)	100% (39.8–100)	Very Low	100% NPV (91.0–100)	54.5%
Intermediate N = 188 (28.2%)				37.3% (27.9–47.4)	90.6% (79.3–96.9)	Low	91.0% NPV (80.8–96.0)	29.4%
	53	102	33					
				94.1% (87.6–97.8)	28.3% (16.8–42.3)	High	65.4% PPV (43.8–82.1)	12.2%
High N = 144 (73.6%)	106	34	4	91.2% (76.3–98.1)	34.0% (25.0–43.8)	Very High	91.5% PPV (77.9–97.0)	27.3%

N, number of patients; including malignant, benign, and clinical benign patients.

Cancer Prevalence is the proportion of malignant patients over total patients (N), including clinical benign.

Specificity is calculated on benign patients only, excluding clinical benign; sensitivity is calculated on malignant patients only.

$PPV=\frac{Prevalence*Sensitivity}{Prevalence*Sensitivity+(1-Prevalence)*(1-Specificity)};\;NPV=\frac{(1-Prevalence)*Specificity}{Prevalence*(1-Sensitivity)+(1-Prevalence)*Specificity}$.

% Reclassified (Low to Very Low, Intermediate to Low) = (1- Prevalence) specificity + Prevalence (1-sensitivity).

% Reclassified (Intermediate to High, High to Very High) = Prevalence ∙ sensitivity + (1-Prevalence) (1- specificity).

NPV (negative predictive value, PPV (positive predictive value), and % Reclassified are all functions of sensitivity, specificity, and cancer prevalence.

Among lesions that were up-classified from intermediate to high ROM, six lesions were benign. These false positives account for 6/102 (5.9%) of all benign intermediate-risk lesions. Five lesions that were down-classified from intermediate to low ROM were malignant. These false negatives account for 5/53 (9.4%) of all malignant intermediate-risk lesions. Three lesions that were classified from high to very high ROM were benign. These false positives account for 3/34 (8.8%) of all benign high-risk lesions. No lesions were falsely down classified from low to very low ROM. NPV and PPV estimates across a range of cancer prevalence (S3 Fig).

A subset of patients in the validation set was identified as having a diagnosis of chronic obstructive pulmonary disease (COPD) based upon the clinical expertise of the investigator at the time of enrollment. In addition to the overall accuracy assessment, the accuracy of the Percepta GSC was assessed for patients with and without COPD. The sensitivity in those with COPD was slightly higher and the specificity slightly lower than those without COPD (S3 Table).

We compared the overall performance of the Percepta GSC using a Receiver Operating Curve (ROC) to provide a comprehensive evaluation of the classifier performance independent of the cut-offs in all three cohorts. We found that the overall performance of the Percepta GSC was similar in the AEGIS I and II cohorts compared to the Percepta Registry with an overall AUC of 0.73 (CI: 68.3–78.4), highlighting the robustness of the classifier performance across different patient cohorts (S4 Table and S4 Fig).

Next, we determined the potential utility of Percepta GSC in decreasing invasive procedure utilization, had the classifier result been available to manage these lesions. Of the 246 patients in the AEGIS I And II cohorts, 241 patients had at least one year of follow-up (S5 Fig). Among the 109 low or intermediate-risk patients with benign lesions, 15 (50%) of 30 patients who underwent additional invasive procedures for benign lesions were down-classified by Percepta GSC and could have potentially avoided these procedures if managed according to the Percepta result (Table 3). Eight of these 15 patients underwent a collective ten surgical procedures. Of the 75 intermediate or high-risk malignant lesions diagnosed as Stage I or II lung cancer, 52 of these patients underwent an intervening diagnostic (not staging) procedure or surveillance CT scan before a surgical resection or ablative therapy. Fifteen (29%) of these 52 patients were up-classified by Percepta GSC and could have avoided an intervening diagnostic procedure if managed according to the Percepta GSC result (Table 4). Overall, 37 intermediate or high-risk malignant lesions were up-classified by Percepta GSC. Of these, 29 (78%) patients were diagnosed with cancer >14 days after the bronchoscopy (turn around time for Percepta GSC is < 14 days) with a median time to diagnosis of 36 days and a maximum time to diagnosis of 739 days. Approximately 1/3 of patients were diagnosed two months after the initial bronchoscopy (S6 Fig).

**Table 3:** Patients* with down-classified low- and intermediate-risk benign lesions who underwent additional invasive procedures.

Pre-Test ROM	Percepta GSC result	Post-test ROM
21 patients (Intermediate-risk Lesion)	Intermediate	13 patients (Intermediate-risk Lesion)
	Low	8 patients (Low-risk Lesion)
9 patients (Low-risk Lesion)	Low	2 patients (Low-risk Lesion)
	Very Low	7 patients (Very Low-risk Lesion)

*Paitents from the AEGIS cohorts only; ROM, risk of malignancy.

**Table 4:** Patients* with intermediate- and high-risk lesions that were up-classified by Percepta GSC and underwent additional procedures prior to malignant diagnosis.

Pre-Test ROM	Percepta GSC result	Post-Test ROM
41 patients (High-risk Lesion)	Very High	13* patients (Very High-Risk Lesion)
	High	28 patients (High-risk Lesion)
11 patients (Intermediate-risk Lesion)	High	3 patients (High-risk Lesion)
	Intermediate	8 patients (Intermediate-risk Lesion)

*Patients from the AEGIS cohorts only; ROM, risk of malignancy; *Unable to verify procedures performed for one patient.

Two patients with malignant lesions who were down-classified from intermediate to low ROM (falsely down-classified) underwent surveillance CT scans or a diagnostic PET scan prior to a diagnosis of malignancy (average time to diagnosis was 8 ± 2 months), suggesting that if the false negative classifier result had been known, it might not have impacted the time to diagnosis. Five patients with benign lesions who were up-classified from intermediate to high ROM underwent six additional procedures, including two surgical resections, one of which occurred 12 days after the bronchoscopy, suggesting that knowledge of the false-positive Percepta GSC result may not have affected the clinical decision to undergo surgical resection.

## Discussion

In this large, three cohort clinical validation study of the second generation Percepta lung nodule classifier, Percepta GSC, the accuracy of the classifier was validated in an independent sample set. High sensitivity with modest specificity for the rule-out portion of the classifier and high specificity with modest sensitivity for the rule-in portion was confirmed. By accurately down-classifying and up-classifying a portion of those with indeterminate lung lesions and a non-diagnostic bronchoscopy, the classifier may influence later management decisions to the patient’s benefit.

With comprehensive machine learning, Percepta GSC can extract relevant genomic signals from transcriptomic sequencing and provide an accurate risk stratification for patients with a non-diagnostic bronchoscopy, one of the most challenging groups in the management of lung lesions. The classifier captures genomic alterations in bronchial epithelial cells collected during bronchoscopy with minimally invasive bronchial brushings [27,28]. Based upon the airway “field of injury” concept that has been previously validated, the classifier could quantify the impact of such genomic alterations on cancer risk, therefore successfully distinguishing malignant nodules from benign lesions [21,22].

The down-classification feature of the classifier enables a reduction in the risk of malignancy with a negative result. In contrast, a positive result confirms the pre-test risk assessment and management decisions. Similarly, the up-classification feature enables an increase in the risk of malignancy with a positive result, while a negative result would confirm pre-test risk assessment and management decisions. Therefore, a portion of those tested will have a test result that could change pre-test clinical management decisions, and a portion will confirm the pre-test management approach.

For those patients with an intermediate pre-test risk lung lesion and a non-diagnostic bronchoscopy, the classifier may be used to down-classify the risk, making the clinician more comfortable with surveillance of the lesion, or to up-classify the risk suggesting additional testing or treatment is warranted. In the population studied within this risk group, the observed sensitivity of 90.6% and specificity of 37.3% for those down-classified led to an actionable negative result in 29.4% of those tested with a ratio of true negative to false-negative results of 10:1. Thus if the test result led to surveillance imaging, ten patients with benign lesions might have avoided further testing, while one patient with a malignant lesion may have had further evaluation delayed. Similarly, for the group whose risk was up-classified from intermediate to high the specificity was 94.1%, with a sensitivity of 28.3%. The observed actionable positive result was 12.2% of those tested with a ratio of true positive to false positive of 1.9:1. Thus if the test result led to more aggressive testing or treatment, approximately two patients with malignant lesions would proceed to additional invasive testing or treatment while one patient with a benign lesion would do the same. Overall, 41.6% of patients with intermediate-risk lesions and non-diagnostic bronchoscopies were classified as a lower or higher risk group.

The ability to risk stratify lesions with low and high pre-test probability of malignancy may lead to greater clinician or patient confidence with management choices. The test characteristics suggest that a negative result from the rule-out feature of the classifier may downgrade the risk of a patient with a low probability lesion and a positive result from the rule-in feature of the classifier may upgrade the risk of a patient with a high probability nodule. In the population studied, 54.5% of low-risk nodules were down-classified to very low risk without any false negatives observed. In comparison, 27.3% of high-risk nodules were up-classified to very high risk with a ratio of true positives to false positives of 12:1. Thus if it resulted in further aggressive therapy, approximately 12 patients with a malignant lesion would be referred for an additional invasive procedure, whereas one patient with a benign lesion would also undergo the same. When the classifier is used across categories of risk (low, intermediate, and high), 39.1% of test results will classify the patient to a category of risk that is different from their pre-test risk category.

Percepta GSC is an enhanced second-generation classifier that demonstrates improved performance characteristics compared to the first-generation BGC. For down-classifying intermediate risk patients, the GSC and BGC performance is similar overall; the point estimate of the NPV for GSC (91.0% [80.8–96.0]) is higher than that for BGC (87.6% NPV [79.0–93.0]) better reducing false negatives, although the difference is not statistically significant. More importantly, GSC has further utility in intermediate risk patients as GSC also may up-classify some intermediate risk patients to high risk. The up-classification of intermediate to high risk patients in GSC occurred in 12.2% of the intermediate risk patients resulting in a post-test high-risk PPV > 65%. Those patients would have received no additional information from the BGC classifier.

The comparison of test accuracy between those with and without COPD provides interesting insight into the nature of the classifier and the field of injury concept. In general, the classifier had a higher sensitivity and lower specificity in those with COPD, whether used as a rule-in or rule-out test. This may suggest biological overlap between genomic changes and COPD and lung cancer clinical features. This knowledge may further increase confidence in negative results in a COPD patient and positive results in those without COPD.

Overall, 27.5% of patients with low- and intermediate-risk lesions that were benign underwent further invasive procedures after a non-diagnostic bronchoscopy. Our study showed that down-classification by Percepta GSC can reduce invasive procedures by 50% in this population. Importantly, patients with nodules that are down-classified by Percepta GSC should be managed according to the guideline recommendation for continued surveillance imaging until the nodule is ascertained to be benign. Given that there may be a small percentage of falsely down-classified malignant lesions, surveillance must be continued until a diagnosis is made or a sufficient duration of follow-up has occurred to confirm benign status. Additionally, the up-classification of intermediate- and high-risk malignant lesions by Percepta GSC would have decreased unnecessary diagnostic procedures in approximately 30% of these patients with the potential for an earlier diagnosis. While these results are promising, they are an estimate of Percepta GSC’s impact on clinical management and, therefore, will need to be confirmed in a “real world” clinical setting. Additional studies will directly answer how often Percepta test results change management decisions, as these decisions are heavily influenced by local treatment patterns and patient values and comorbidities.

Strengths of the study include use of three large, independent multicenter cohorts, which included patients from different types of clinical practices across several geographical locations to assess clinical accuracy metrics of the Percepta GSC. Additionally, the classifier was validated using a locked classifier after completion of algorithm development and technical validation phases. This updated classifier extends the range of potential utility by adding a rule-in component to the test for patients with a pre-test intermediate-risk lung lesion. Finally, this clinical validation of the Percepta GSC was performed in patients with a non-diagnostic bronchoscopy, thus reflecting the population where the test will have potential utility.

Limitations of the results include the adjudication process where follow-up was only required to be 12 months to determine benign status. This may have contributed to the inability to adjudicate a diagnosis for 45 patients (not included in the sensitivity and specificity metrics but used to estimate prevalence assuming benignity). Thus a few indolent lung cancers could have been present, and the true prevalence of malignancy may have been slightly higher. It is unclear whether identifying indolent malignancies would impact the utility of the classifier, as surveillance of indolent malignancies is less likely to influence outcomes. Additional prospective clinical utility studies would be helpful to further establish the benefits and performance of the classifier in real-world settings.

As is true with all risk of malignancy prediction models, shifts from one risk category to another are based on negative and positive predictive values, the calculation of which requires the prevalence of malignancy within those risk groups. This study utilized three independent cohorts to establish cancer prevalence at each risk level; however, prevalence may vary in an individual clinical practice. To assist with the application of the test, we provided figures showing post-test probabilities across a range of pre-test probabilities in the supplement, assuming consistent sensitivity and specificity across all pre-test ROMs (S3A–S3D Fig).

Finally, results presented here describe the evaluation of the clinical validity of Percepta GSC and are not intended to provide a comprehensive assessment of clinical utility. Clinician decisions are based not only on the probability of malignancy but also on the accuracy and safety of available testing, patient comorbidities, and patient preferences. This clinical validation study confirmed the accuracy of the Percepta GSC, showing high sensitivity for the rule-out portion of the classifier and high specificity for the rule-in portion of the classifier. Use of the classifier could impact clinical decisions in up to 40% of patients with lung lesions and indeterminate results from bronchoscopy. Further assessment of clinical utility is warranted.

## Supporting information

S1 Fig Study population for the AEGIS I and II. Consort diagram of the derivation of the study population from the AEGIS I and II cohorts for this validation study.
(TIF)

S2 Fig Study population for the Percepta Registry. Consort diagram of the derivation of the study population from the Percepta Registry cohort for this validation study.
(TIF)

S3 Fig Negative and Positive Predictive Value (NPV) of Percepta GSC across cancer prevalence. a) Negative predictive value (NPV) of the Percepta GSC across different pre-test cancer prevalence in patients who are classified from low to very low risk with specificity of 57.4% and sensitivity of 100%. The prevalence of lung cancer with and without these 45 clinically benign patients was 5.0% and 5.6% in the low pre-test ROM group, respectively. b) Negative predictive value (NPV) of the Percepta GSC across different pre-test cancer prevalence in patients who are classified from intermediate to low risk with specificity of 37.3% and sensitivity of 90.6%. The prevalence of lung cancer with and without these 45 clinically benign patients was 28.2% and 34.2% in the intermediate pre-test ROM group, respectively. c) Positive predictive value (PPV) of the Percepta GSC across different pre-test cancer prevalence in patients who are classified from intermediate to high risk with specificity of 94.1% and sensitivity of 28.3%. The prevalence of lung cancer with and without these 45 clinically benign patients was 28.2% and 34.2% in the intermediate pre-test ROM group, respectively. d) Positive predictive value (PPV)of the Percepta GSC across different pre-test cancer prevalence in patients who are classified from high to very high risk with specificity of 91.2% and sensitivity of 34.0%. The prevalence of lung cancer with and without these 45 clinically benign patients was 73.6% and 75.7% in the high pre-test ROM group, respectively.
(TIF)

S4 Fig Percepta GSC performance in the AEGIS I and II and Percepta Registry cohorts. a) Comparison of the receiver operator curve (ROC) of the Percepta GSC in all study patients in the AEGIS I and II cohorts and the Percepta Registry. b) Comparison of the receiver operator curve (ROC) of the Percepta GSC in the low and intermediate risk of malignancy study patients in the AEGIS I and II cohorts and the Percepta Registry. The asterisk on each curve corresponds to the sensitivity/specificity pair at the decision boundary where patients with scores above the decision boundary will maintain their risk of malignancy; and patients with scores below the decision boundary will have their risk of malignancy down-classified (i.e. low to very low and intermediate to low). c) Comparison of the receiver operator curve (ROC) of the Percepta GSC in the intermediate risk of malignancy study patients in the AEGIS I and II cohorts and the Percepta Registry. The asterisk on each curve corresponds to the sensitivity/specificity pair at the decision boundary where patients with scores above the decision boundary will have their risk malignancy up-classified from intermediate to high; and patients with scores below the decision boundary will have their risk of malignancy stay as intermediate. d) Comparison of the receiver operator curve (ROC) of the Percepta GSC in the high risk of malignancy study patients in the AEGIS I and II cohorts and the Percepta Registry. The asterisk on each curve corresponds to the sensitivity/specificity pair at the decision boundary where patients with scores above the decision boundary will have their risk malignancy up-classified from high to very high; and patients with scores below the decision boundary will have their risk of malignancy stay as high.
(TIF)

S5 Fig Summary of procedures among patients in the AEGIS I/II cohorts. Consort diagram of patients in the AEGIS cohorts included in the clinical validation of Percepta GSC. The flow chart depicts the number of patients with benign and malignant lesions who had a pre-test ROM and underwent additional procedures for an indeterminate lung lesion after a non-diagnostic bronchoscopy and the number of patients who were risk reclassified by Percepta GSC.
(TIF)

S6 Fig Time to malignant diagnosis in 37 up-classified intermediate and high risk nodules in the AEGIS I/II cohorts. Bar chart shows the time to malignant diagnoses in 37 up-classified intermediate- and high-risk lesions in the AEGIS I/II cohorts. A total of 29 (78%) patients were diagnosed with cancer >14 days after the bronchoscopy, with a median time to diagnosis of 36 days and a maximum time to diagnosis of 739 days. A subset of 12 (32.4%) patients were diagnosed two months after the initial bronchoscopy.
(TIF)

S1 Table AEGIS I and II cohort sites and investigators. (DOCX)

S2 Table Percepta Registry sites and investigators. (DOCX)

S3 Table Percepta GSC performance in patients in AEGIS I and II and Percepta Registry cohorts. (DOCX)

S4 Table Percepta GSC performance in subset of patients with and without COPD. (DOCX)

S5 Table Total number of additional procedures performed in patients with down-classified low and intermediate risk benign lesions. (DOCX)

S6 Table Total number of additional procedures performed in patients with up-classified intermediate and high risk lesions prior to malignant diagnosis. (DOCX)

S1 Text Laboratory methods: mRNA processing and sequencing. (DOCX)

S2 Text Percepta GSC algorithm development. (DOCX)
