# Supplementary figures and images for: Clinical validation and utility of Percepta GSC for the evaluation of lung cancer
Source: PLoS One. 2022 Jul 13;17(7):e0268567. doi: 10.1371/journal.pone.0268567 (PMC9278743; doi:10.1371/journal.pone.0268567)

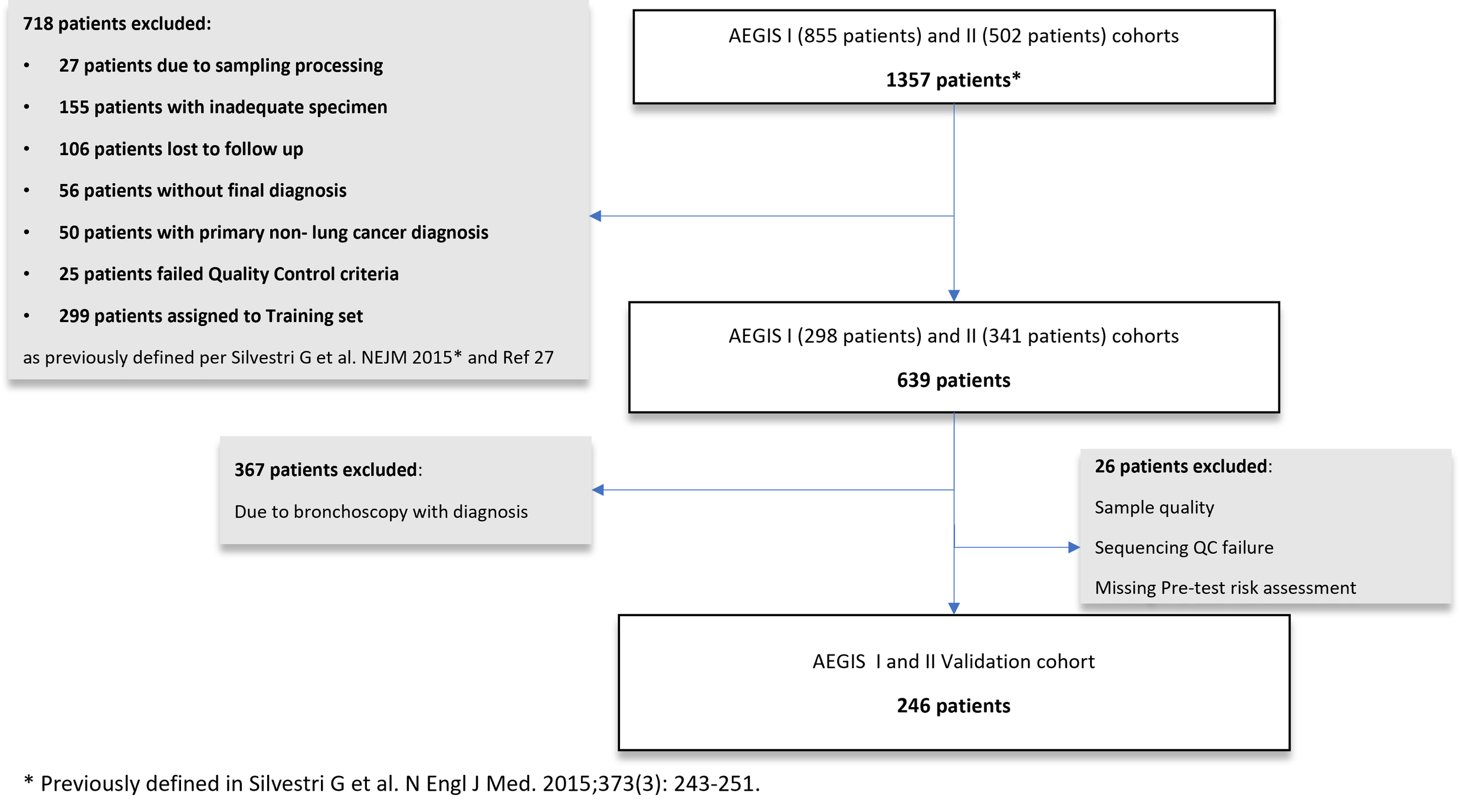

Supplement: S1 Fig — Consort diagram of the derivation of the study population from the AEGIS I and II cohorts for this validation study. (TIF) [file pone.0268567.s001.tif]

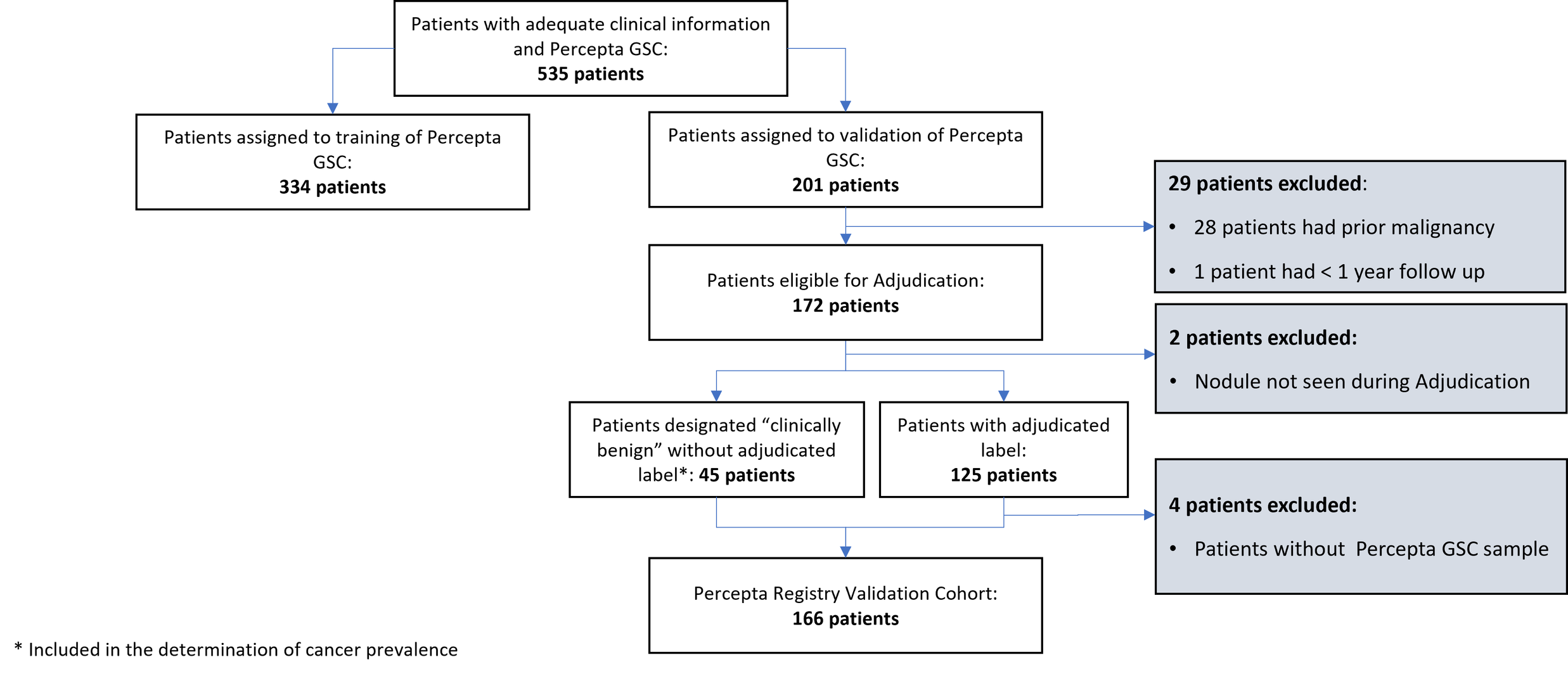

Supplement: S2 Fig — Consort diagram of the derivation of the study population from the Percepta Registry cohort for this validation study. (TIF) [file pone.0268567.s002.tif]

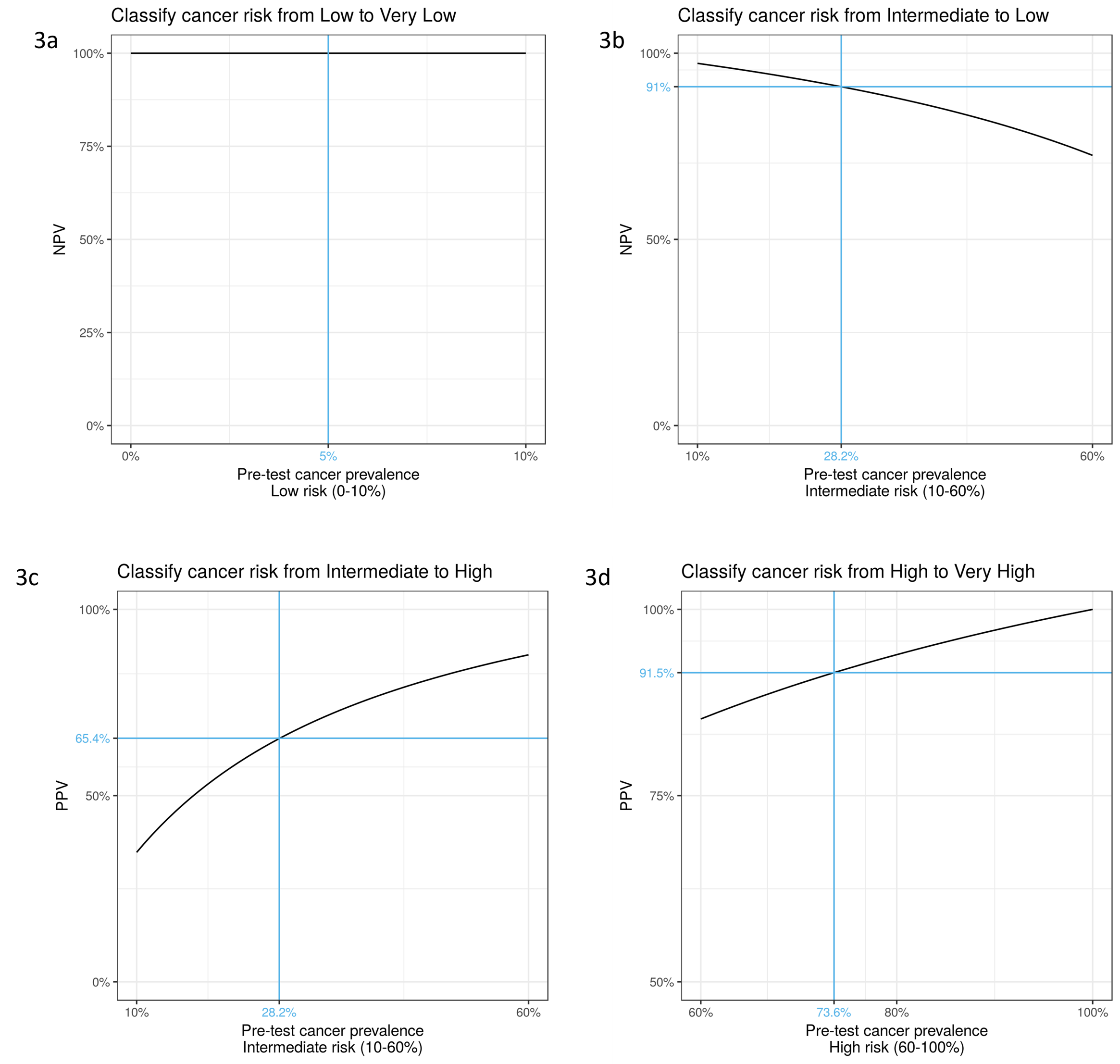

Supplement: S3 Fig — a) Negative predictive value (NPV) of the Percepta GSC across different pre-test cancer prevalence in patients who are classified from low to very low risk with specificity of 57.4% and sensitivity of 100%. The prevalence of lung cancer with and without these 45 clinically benign patients was 5.0% and 5.6% in the low pre-test ROM group, respectively. b) Negative predictive value (NPV) of the Percepta GSC across different pre-test cancer prevalence in patients who are classified from intermediate to low risk with specificity of 37.3% and sensitivity of 90.6%. The prevalence of lung cancer with and without these 45 clinically benign patients was 28.2% and 34.2% in the intermediate pre-test ROM group, respectively. c) Positive predictive value (PPV) of the Percepta GSC across different pre-test cancer prevalence in patients who are classified from intermediate to high risk with specificity of 94.1% and sensitivity of 28.3%. The prevalence of lung cancer with and without these 45 clinically benign patients was 28.2% and 34.2% in the intermediate pre-test ROM group, respectively. d) Positive predictive value (PPV)of the Percepta GSC across different pre-test cancer prevalence in patients who are classified from high to very high risk with specificity of 91.2% and sensitivity of 34.0%. The prevalence of lung cancer with and without these 45 clinically benign patients was 73.6% and 75.7% in the high pre-test ROM group, respectively. (TIF) [file pone.0268567.s003.tif]

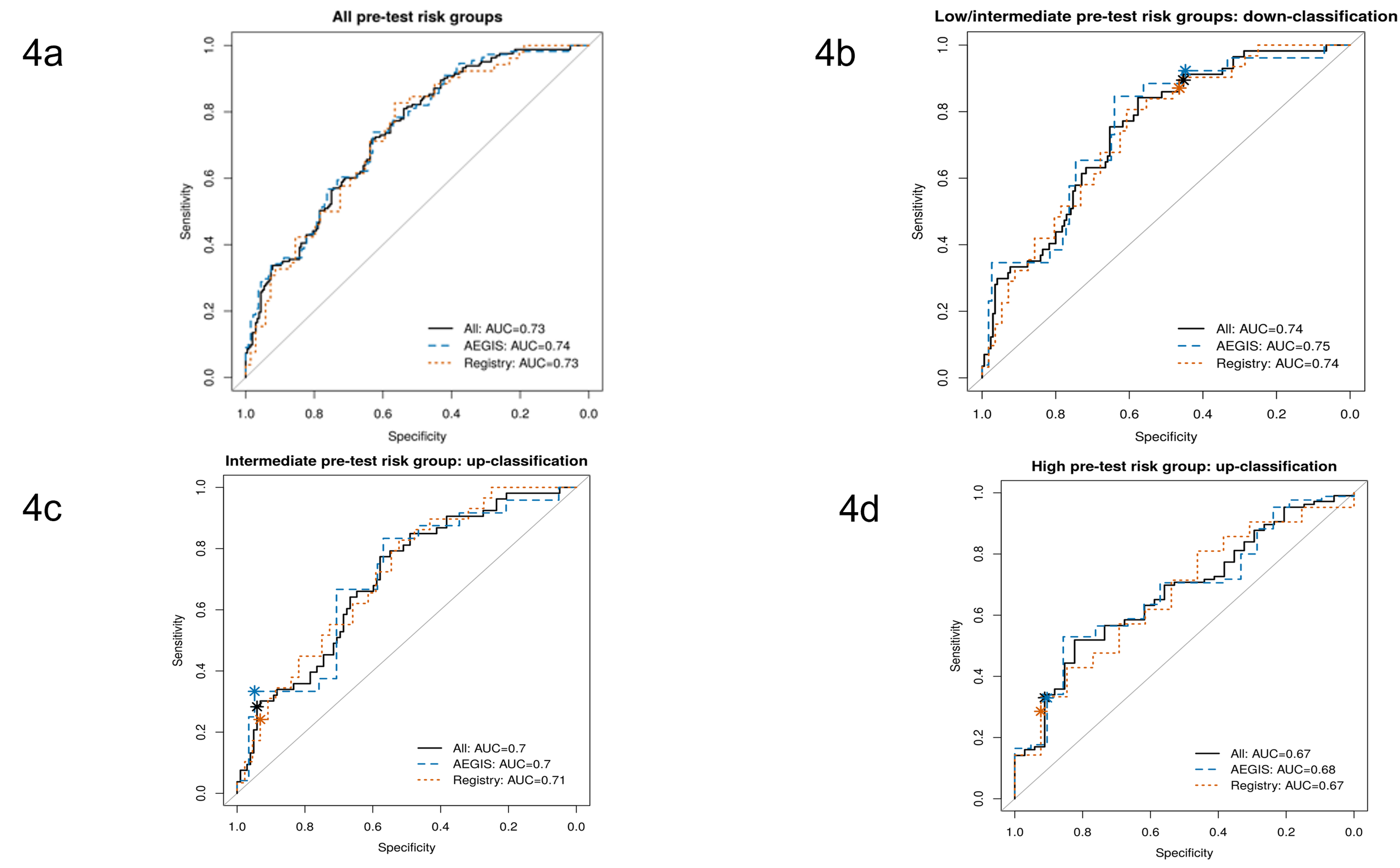

Supplement: S4 Fig — a) Comparison of the receiver operator curve (ROC) of the Percepta GSC in all study patients in the AEGIS I and II cohorts and the Percepta Registry. b) Comparison of the receiver operator curve (ROC) of the Percepta GSC in the low and intermediate risk of malignancy study patients in the AEGIS I and II cohorts and the Percepta Registry. The asterisk on each curve corresponds to the sensitivity/specificity pair at the decision boundary where patients with scores above the decision boundary will maintain their risk of malignancy; and patients with scores below the decision boundary will have their risk of malignancy down-classified (i.e. low to very low and intermediate to low). c) Comparison of the receiver operator curve (ROC) of the Percepta GSC in the intermediate risk of malignancy study patients in the AEGIS I and II cohorts and the Percepta Registry. The asterisk on each curve corresponds to the sensitivity/specificity pair at the decision boundary where patients with scores above the decision boundary will have their risk malignancy up-classified from intermediate to high; and patients with scores below the decision boundary will have their risk of malignancy stay as intermediate. d) Comparison of the receiver operator curve (ROC) of the Percepta GSC in the high risk of malignancy study patients in the AEGIS I and II cohorts and the Percepta Registry. The asterisk on each curve corresponds to the sensitivity/specificity pair at the decision boundary where patients with scores above the decision boundary will have their risk malignancy up-classified from high to very high; and patients with scores below the decision boundary will have their risk of malignancy stay as high. (TIF) [file pone.0268567.s004.tif]

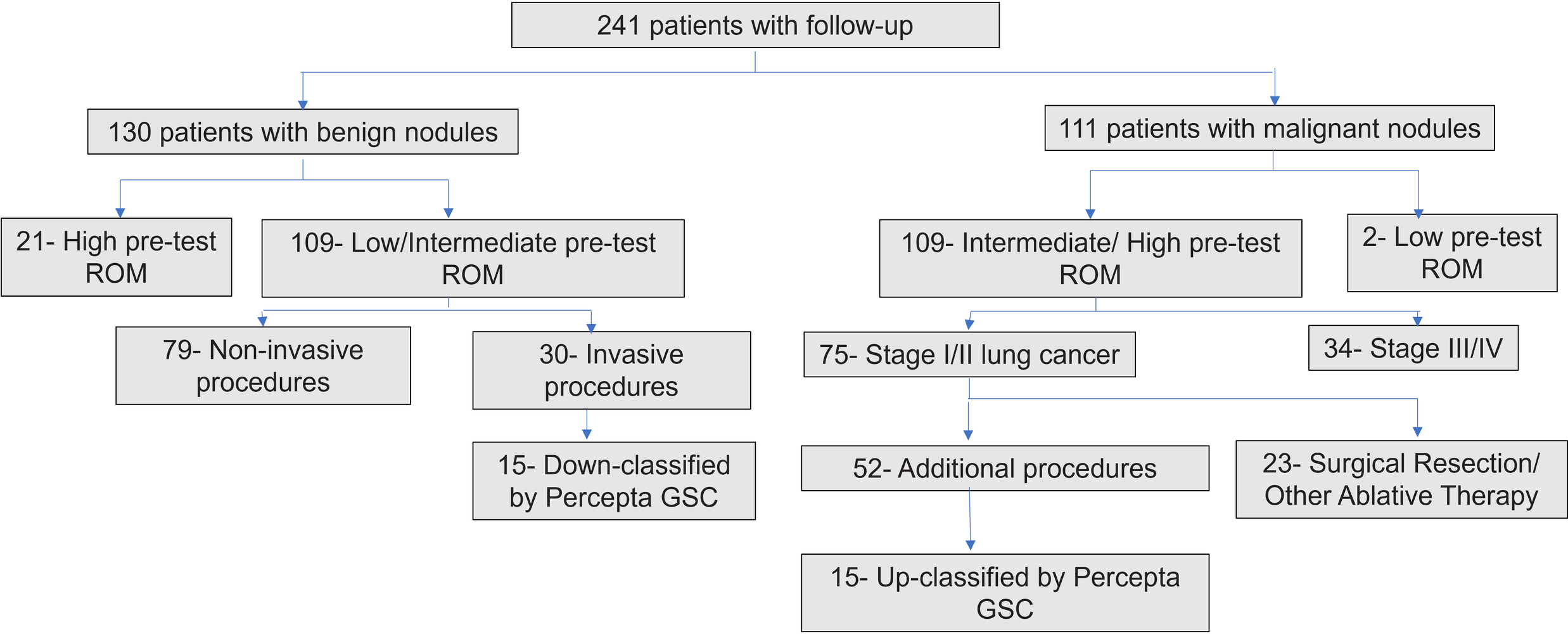

Supplement: S5 Fig — Consort diagram of patients in the AEGIS cohorts included in the clinical validation of Percepta GSC. The flow chart depicts the number of patients with benign and malignant lesions who had a pre-test ROM and underwent additional procedures for an indeterminate lung lesion after a non-diagnostic bronchoscopy and the number of patients who were risk reclassified by Percepta GSC. (TIF) [file pone.0268567.s005.tif]

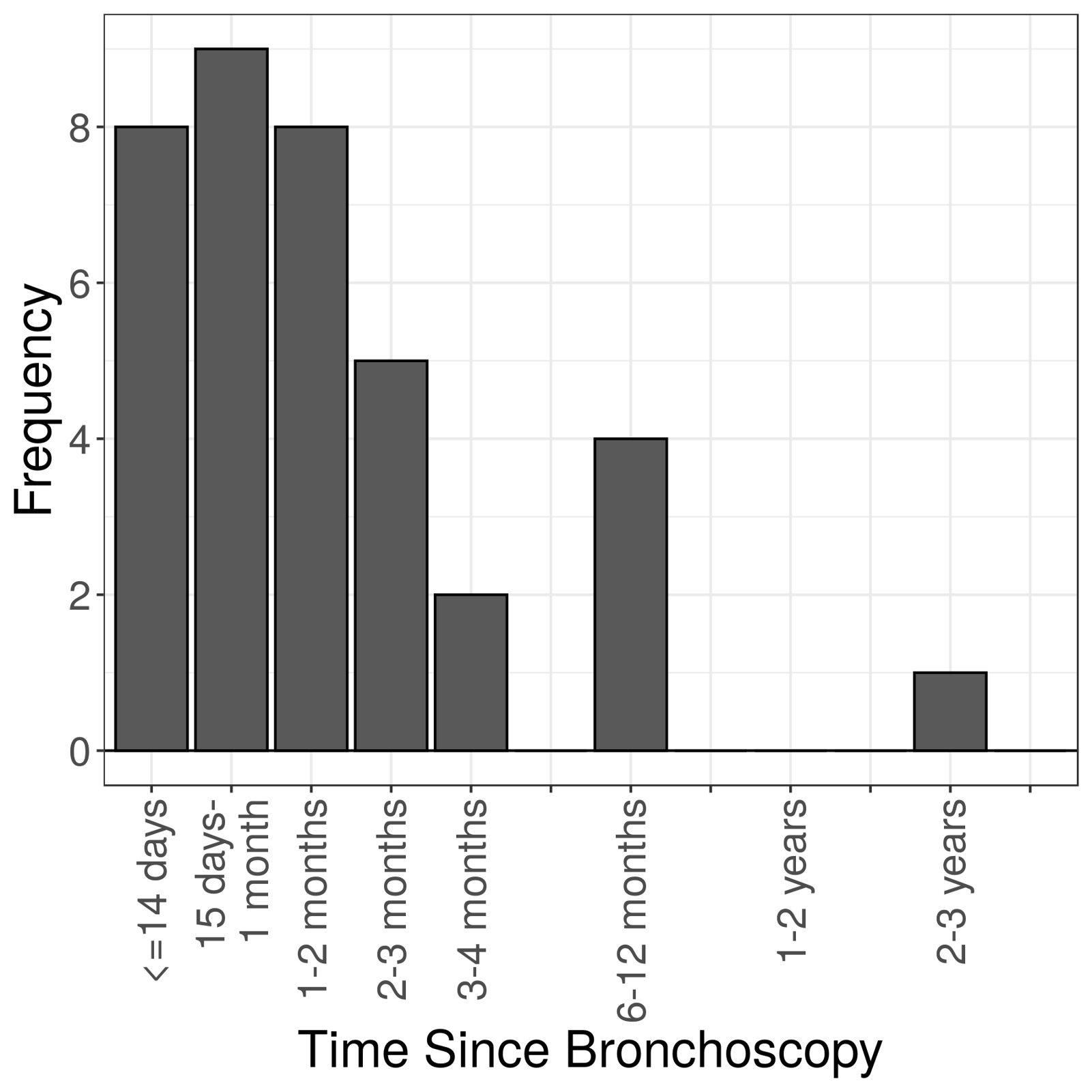

Supplement: S6 Fig — Bar chart shows the time to malignant diagnoses in 37 up-classified intermediate- and high-risk lesions in the AEGIS I/II cohorts. A total of 29 (78%) patients were diagnosed with cancer >14 days after the bronchoscopy, with a median time to diagnosis of 36 days and a maximum time to diagnosis of 739 days. A subset of 12 (32.4%) patients were diagnosed two months after the initial bronchoscopy. (TIF) [file pone.0268567.s006.tif]
