# Supplementary material for: Clinical validation and utility of Percepta GSC for the evaluation of lung cancer
Source: PLoS One. 2022 Jul 13;17(7):e0268567. doi: 10.1371/journal.pone.0268567 (PMC9278743; doi:10.1371/journal.pone.0268567)
Supplement: S1 Table — (DOCX) [file pone.0268567.s007.docx]

**S1 Table. AEGIS I and II Cohort Sites and Investigators**

| **Participating Center** | **Site Location** | **PI Name** |
| --- | --- | --- |
| Beth Israel Deaconess Medical Center | Boston, MA | Armin Ernst and Gaetane Michaud |
| University of British Columbia | Vancouver, BC, Canada | Stephen Lam |
| University of Pennsylvania | Philadelphia, PA | Anil Vachani |
| New York University | New York, NY | William Rom |
| Temple University | Philadelphia, PA | John Travaline |
| Indiana University | Indianapolis, IN | Francis Sheski |
| University of Virginia | Charlottesville, VA | George Verghese |
| University of Missouri | Columbia, MO | Vamsi Guntur |
| Louisiana State University | New Orleans, LA | Stephen Kantrow |
| St. James’s Hospital, Trinity College | Dublin, Ireland | Joe Keane |
| Georgia Clinical Research | Austell, GA | Stuart Simon |
| University of Wisconsin | Madison, WI | Scott Ferguson |
| Medical University of South Carolina | Charleston, SC | Gerard Silverstri |
| National Jewish Health | Denver, CO | Ali Musani |
| Overlake Hospital | Bellevue, WA | Amy Markezich |
| Pulmonary Associates, P.A. | Phoenix, AZ | Mark Gotfried |
| William Jennings Bryan Dorn VAMC | Columbia, SC | Brian Smith and Andrea Mass |
| Virginia Commonwealth University | Richmond, VA | Wes Shepherd |
| Jamaica Hospital Medical Center | Jamaica, NY | Craig Thurm |
| University of California- Davis | Sacramento, CA | Richart Harper |
| North Florida/South Georgia Veterans Health System | Gainesville, FL | Peruvemba Sriram |
| St. Elizabeth's Medical Center | Brighton, MA | Samaan Rafeq and Armin Ernst |
| Yale | New Haven, CT | Gaetane Michaud |
| Cleveland Clinic | Cleveland, OH | Tom Gildea |
