# Supplementary material for: Clinical validation and utility of Percepta GSC for the evaluation of lung cancer
Source: PLoS One. 2022 Jul 13;17(7):e0268567. doi: 10.1371/journal.pone.0268567 (PMC9278743; doi:10.1371/journal.pone.0268567)
Supplement: S2 Table — (DOCX) [file pone.0268567.s008.docx]

**S2 Table. Percepta Registry Sites and Investigators**

| **Participating Center** | **Site Location** | **PI Name** |
| --- | --- | --- |
| Baptist Health Louisville | Louisville, KY | Mark Esterle |
| Blount Memorial Hospital | Alcoa, TN | Gregory LeMense, Tyler Bowen |
| Central Baptist Health | Lexington, KY | Patton Thompson |
| Cooper Health | Camden, NJ | Wissam Abouzgheib |
| Duke University | Durham, NC | Momen Wahidi |
| Gundersen Clinic | La Crosse, WI | Julio Bird, Jennifer Mattingley |
| Illinois Lung and Critical Care Institute | Peoria, IL | Patrick Whitten |
| Kettering Medical Center | Kettering, OH | Ehab Hussein, Hemant Shah |
| Lahey Hospital and Medical Center | Burlington, MA | Carla Lamb |
| Medical College of Wisconsin | Milwaukee, WI | Ali Musani, Vijaya Ramalingam |
| Medical University of South Carolina | Charleston, SC | Gerard Silverstri |
| CHI Memorial Hospital | Chattanooga, TN | Krish Bhadra |
| Pinehurst Medical Center | Pinehurst, NC | Michael Pritchett |
| Pueblo Pulmonary Associates | Pueblo, CO | Joshiah Gordon |
| Pulmonary Consultants | Colorado Springs, CO | Alain Eid |
| PulmonIx | Greensboro, NC | Robert Byrum |
| Ralph H. Johnson Veteran Affairs Medical Center | Charleston, SC | Nichole Tanner |
| Robert J. Dole VA | Wichita, KS | Jing Liu |
| Rutgers | New Brunswick, NJ | Sugeet Jagpal |
| Schneck Medical Center | Seymour, IN | David Wilson |
| Stamford Hospital | Stamford, CT | Michael Berstein |
| Stanford University | Palo Alto, CA | Arthur Sung |
| The Cleveland Clinic | Cleveland, OH | Peter Mazzone |
| The Johns Hopkins Hospital | Baltimore, MD | Hans Lee |
| University of Alabama at Birmingham | Birmingham, AL | Hitesh Batra |
| University of Chicago | Chicago, IL | Kyle Hogarth, Septimiu Murgu |
| University of Cincinnati | Cincinnati, OH | Sadia Benzaquen |
| University of Louisville | Louisville, KY | Tanya Wiese, Umair Gauhar |
| University of Maryland | Baltimore, MD | Ashutosh Sachdeva |
| University of North Carolina | Chapel Hill, NC | Jason Akulian, Adam Belanger |
| University of Wisconsin at Madison | Madison, WI | J. Scott Ferguson |
| UT Health Athens | Tyler, TX | David Gass |
| Wake Forest Baptist Medical Center | Winston-Salem, NC | Travis Dotson |
| Waterbury Pulmonary Associates | Waterbury, CT | David Hill |
