# Supplementary material for: Clinical validation and utility of Percepta GSC for the evaluation of lung cancer
Source: PLoS One. 2022 Jul 13;17(7):e0268567. doi: 10.1371/journal.pone.0268567 (PMC9278743; doi:10.1371/journal.pone.0268567)
Supplement: S3 Table — (DOCX) [file pone.0268567.s009.docx]

**S3 Table. Percepta GSC performance in patients in AEGIS I and II and Percepta Registry Cohorts**

| **Pre-test**  **Cancer Risk** | **AEGIS I and II** | | | **Percepta Registry** | | |
| --- | --- | --- | --- | --- | --- | --- |
|  | **N** | **Specificity** | **Sensitivity** | **N** | **Specificity** | **Sensitivity** |
| Low | 58 | 55.4%  (41.5 – 68.7) | 100%  (15.8 – 100) | 14 | 100%  (2.5 – 100) | 100%  (2.5 – 100) |
| Intermediate | 82 | 34.5%  (22.5 – 48.1) | 91.7%  (73.0 – 99.0) | 73 | 40.9%  (26.3 – 56.8) | 89.7%  (72.6 – 97.8) |
|  |  | 94.8%  (85.6 – 98.9) | 33.3%  (15.6 – 55.3) |  | 93.2%  (81.3 – 98.6) | 24.1%  (10.3 – 43.5) |
| High | 106 | 90.5%  (69.6 – 98.8) | 34.1%  (24.2 – 45.2) | 34 | 92.3%  (64.0 – 99.8) | 33.3%  (14.6 – 57.0) |

AEGIS, Airway Epithelium Gene Expression in the Diagnosis of Lung Cancer; N, number of patients; 95% confidence intervals in parentheses.
