# Supplementary material for: Clinical validation and utility of Percepta GSC for the evaluation of lung cancer
Source: PLoS One. 2022 Jul 13;17(7):e0268567. doi: 10.1371/journal.pone.0268567 (PMC9278743; doi:10.1371/journal.pone.0268567)
Supplement: S4 Table — (DOCX) [file pone.0268567.s010.docx]

**S4 Table. Percepta GSC performance in subset of patients with and without COPD**

| **Pre-test**  **Cancer Risk** |  | **COPD** | | | **non-COPD** | | |
| --- | --- | --- | --- | --- | --- | --- | --- |
|  | **Percepta GSC result** | **N** | **Specificity** | **Sensitivity** | **N** | **Specificity** | **Sensitivity** |
| Low | Very Low | 18 | 35.3%  (14.2 – 61.7) | 100%  (2.5 – 100) | 54 | 64.7%  (50.1 – 77.6) | 100%  (29.2 – 100) |
| Intermediate | Low | 54 | 18.2%  (7.0 – 35.5) | 95.2%  (76.2 – 99.9) | 101 | 46.4%  (34.3 – 58.8) | 87.5%  (71 – 96.5) |
|  | High |  | 90.9%  (75.7 – 98.1) | 47.6%  (25.7 – 70.2) |  | 95.7%  (87.8 – 99.1) | 15.6%  (5.3 – 32.8) |
| High | Very High | 64 | 88.9%  (51.8 – 99.7) | 45.5%  (32.0 – 59.4) | 76 | 92.0%  (74.0 – 99.0) | 21.6%  (11.3 – 35.3) |

N, number of patients; COPD, chronic obstructive pulmonary disease; 95% confidence intervals in parentheses.
