# Supplementary material for: Clinical validation and utility of Percepta GSC for the evaluation of lung cancer
Source: PLoS One. 2022 Jul 13;17(7):e0268567. doi: 10.1371/journal.pone.0268567 (PMC9278743; doi:10.1371/journal.pone.0268567)
Supplement: S5 Table — (DOCX) [file pone.0268567.s011.docx]

**S5 Table. Total number of additional procedures performed in patients with down-classified low and intermediate risk benign lesions**

|  | All | Low to Very Low Risk (<1%) by Percepta GSC | Intermediate to Low Risk (< 10%) by Percepta GSC |
| --- | --- | --- | --- |
| **Total procedures** | 25 | 10 | 15 |
| Surgery | 10 | 4 | 6 |
| TTNA/B | 3 | 1 | 2 |
| Bronchoscopy | 12 | 5 | 7 |

TTNA/B, trans-thoracic needle aspiration or biopsy
