# Supplementary material for: Clinical validation and utility of Percepta GSC for the evaluation of lung cancer
Source: PLoS One. 2022 Jul 13;17(7):e0268567. doi: 10.1371/journal.pone.0268567 (PMC9278743; doi:10.1371/journal.pone.0268567)
Supplement: S6 Table — (DOCX) [file pone.0268567.s012.docx]

**S6 Table. Total number of additional procedures performed in patients with up-classified intermediate and high risk lesions prior to malignant diagnosis**

|  | All | High to Very High Risk (>95%)  by Percepta GSC | Intermediate to High Risk (>65%)  by Percepta GSC |
| --- | --- | --- | --- |
| **Invasive Procedures** | **14** | **12** | **2** |
| TTNA/B | 12 | 10 | 2 |
| Bronchoscopy | 2 | 2 | 0 |
| **Non-Invasive Procedures** | **4** | **1** | **3** |
| CT | 2 | 1 | 1 |
| PET | 2 | 0 | 2 |

TTNA/B, trans-thoracic needle aspiration or biopsy
