# Supplementary material for: Clinical validation and utility of Percepta GSC for the evaluation of lung cancer
Source: PLoS One. 2022 Jul 13;17(7):e0268567. doi: 10.1371/journal.pone.0268567 (PMC9278743; doi:10.1371/journal.pone.0268567)
Supplement: S1 Text — (DOCX) [file pone.0268567.s013.docx]

**Methods**

**Laboratory Methods: mRNA Processing and Sequencing**

Two bronchial brush specimens were collected from the normal-appearing right mainstem bronchus during bronchoscopy, stored in a nucleic acid preservative (RNAprotect, QIAGEN, Hilden, Germany), then shipped (2-8 ℃) to the testing laboratory. From each brushing sample, total RNA was extracted using the miRNeasy Mini Kit (QIAGEN, Hilden, Germany), quantitated (QuantiFluor RNA System, Promega, Madison, WI) and 50ng was used as input to the TruSeq RNA Access Library Prep procedure (Illumina, San Diego, CA) for coding transcriptome enrichment. Libraries meeting quality control criteria were sequenced using NextSeq 500 instruments (2x75 bp paired-end reads) with the High Output Kit (Illumina, San Diego, CA). Raw sequencing (FASTQ) files were aligned to the Human Reference assembly 37 (Genome Reference Consortium) using the STAR RNA-seq aligner software [9]. Uniquely mapped and non-duplicate reads were summarized for 63,677 annotated Ensembl genes using HTSeq [10]. Data quality metrics were generated using RNA-SeQC [11]. Samples were excluded and re-sequenced when their library sequence data did not achieve minimum criteria for total reads, uniquely mapped reads, mean per-base coverage, base duplication rate, percentage of bases aligned to coding regions, base mismatch rate, and uniformity of coverage within each gene.
