# Supplementary material for: Clinical validation and utility of Percepta GSC for the evaluation of lung cancer
Source: PLoS One. 2022 Jul 13;17(7):e0268567. doi: 10.1371/journal.pone.0268567 (PMC9278743; doi:10.1371/journal.pone.0268567)
Supplement: S2 Text — (DOCX) [file pone.0268567.s014.docx]

**Percepta GSC Algorithm Development**

Normalization and gene filtering of the genomic sequencing data and the derivation of the algorithm of the Percepta GSC in the training cohort was previously described (27). The final ensemble score from the Percepta GSC algorithm is the logit of mean probabilities from four individual models. Together, the final ensemble classifier includes five clinical features (age, gender, pack-year, inhaled medication use, and specimen collection timing) and 1,232 gene features. This final ensemble classifier was developed and prospectively locked on a prior training cohort (27). With the same underlying classifier, Percepta GSC deploys multiple thresholds allowing it to serve as both a “rule in” and a “rule out” test for different pre-test risk groups (27). Specifically, the first threshold is used to down-classify patients with pre-test intermediate risk to low risk as well as down-classify patients with pre-test low risk to very low risk when they are below that threshold value (figure E4b); the second threshold is used to up-classify patients with pre-test intermediate risk to high risk when they are above that threshold value (Figure E4c); and the last threshold is used to up-classify patients with pre-test high risk to very high risk when they are above that threshold value (Figure E4d). This multiple-thresholds approach increases the potential utility of Percepta GSC in improving risk stratification (27).
